# Increased Postprandial Energy Expenditure May Explain Superior Long Term Weight Loss after Roux-en-Y Gastric Bypass Compared to Vertical Banded Gastroplasty

**DOI:** 10.1371/journal.pone.0060280

**Published:** 2013-04-03

**Authors:** Malin Werling, Torsten Olbers, Lars Fändriks, Marco Bueter, Hans Lönroth, Kaj Stenlöf, Carel W. le Roux

**Affiliations:** 1 Department of Gastrosurgical Research and Education, Sahlgrenska Academy, University of Gothenburg, Sahlgrenska University Hospital/Sahlgrenska, Gothenburg, Sweden; 2 Department of Surgery, Division of Visceral and Transplantation Surgery, University Hospital Zurich, Zurich, Switzerland; 3 Department of Clinical Research, Sahlgrenska University Hospital, Gothenburg, Sweden; 4 Experimental Pathology, UCD Conway Institute, School of Medicine and Medical Sciences, University College Dublin, Dublin, Ireland; Scientific Directorate, Bambino Hospital, Italy

## Abstract

**Background and Aims:**

Gastric bypass results in greater weight loss than Vertical banded gastroplasty (VBG), but the underlying mechanisms remain unclear. In addition to effects on energy intake the two bariatric techniques may differentially influence energy expenditure (EE). Gastric bypass in rats increases postprandial EE enough to result in elevated EE over 24 hours. This study aimed to investigate alterations in postprandial EE after gastric bypass and VBG in humans.

**Methods:**

Fourteen women from a randomized clinical trial between gastric bypass (n = 7) and VBG (n = 7) were included. Nine years postoperatively and at weight stability patients were assessed for body composition and calorie intake. EE was measured using indirect calorimetry in a respiratory chamber over 24 hours and focused on the periods surrounding meals and sleep. Blood samples were analysed for postprandial gut hormone responses.

**Results:**

Groups did not differ regarding body composition or food intake either preoperatively or at study visit. Gastric bypass patients had higher EE postprandially (p = 0.018) and over 24 hours (p = 0.048) compared to VBG patients. Postprandial peptide YY (PYY) and glucagon like peptide 1 (GLP-1) levels were higher after gastric bypass (both p<0.001).

**Conclusions:**

Gastric bypass patients have greater meal induced EE and total 24 hours EE compared to VBG patients when assessed 9 years postoperatively. Postprandial satiety gut hormone responses were exaggerated after gastric bypass compared to VBG. Long-term weight loss maintenance may require significant changes in several physiological mechanisms which will be important to understand if non-surgical approaches are to mimic the effects of bariatric surgery.

## Introduction

The worldwide obesity epidemic continues undiminished, resulting in increased morbidity and mortality [1], [2] with serious personal, social, and economic consequences [3]. While non-surgical therapies are currently of limited efficacy [4], bariatric surgery provides long term body weight loss and maintenance associated with decreased morbidity and mortality [5]–[7].

The magnitude of weight loss and metabolic effects are not uniform and show a wide distribution between standard bariatric procedures. For example, gastric bypass has superior long term weight loss maintenance and resolution of co morbidities compared to vertical banded gastroplasty (VBG) [6], [8]. The mechanisms remain to be fully elucidated, but one of the differences is the increased postprandial levels of glucagon-like peptide 1 (GLP-1) and peptide YY (PYY) associated with enhanced satiation after gastric bypass [9], [10]. The exaggerated GLP-1 or PYY responses after food intake are absent after VBG. Release of GLP-1 and of PYY from endocrine L cells in the distal ileum and colon are attenuated in morbidly obese subjects after food intake [11], while exaggerated after gastric bypass [12]. In contrast to gastric bypass, patients after VBG usually do not report long lasting changes in appetite. Weight loss after VBG, seems primarily due to a narrowed gastric lumen restraining patients from ingesting large meals leading to lowered total calorie intake [13], [14], although food preferences tend to change towards energy rich foods [14].

Until now, weight loss after bariatric operations has been mainly attributed to reduced food ingestion, whereas effects on energy expenditure have not been fully explored. Diurnal energy expenditure is positively related to body weight and fat free mass [15]. Hence, obese weight-stable individuals have a greater total energy turnover compared to lean individuals. Three major factors account for this observation; 1. the body mass *per se* and particularly the greater amount of lean body mass, both of which consume energy; 2. the postprandial energy expenditure which is higher due to greater food intake and 3. increased energy cost of activities of daily living while having a greater body weight [16]. Consequently, reduced energy expenditure is a physiological response which may promote weight regain [17]–[21]. This antagonizes long-term weight loss after diets and at least partly explains why dietary interventions often are unsuccessful in maintaining long-term weight loss.

Similar to diet induced weight loss, patients after gastric bypass and VBG have a decrease in resting energy expenditure as a function of the amount of weight lost [18], [22], [23]. However, animal experiments indicate that the pattern of energy expenditure might be different after gastric bypass surgery. We and others demonstrated increased diet-induced energy expenditure in rats after gastric bypass operations compared to sham operated, body weight matched rats [24], [25]. The diet-induced effects in rats were sufficient to increase 24 hour energy expenditure even though no changes were observed in physical activity or body temperature between groups [24], [25]. However, the gastric bypass effects on energy expenditure in humans are controversial with some studies reporting increases and others no change or decreases of energy expenditure [23], [26]–[28]. Discrepancies may be at least partly explained by heterogeneous patient populations and/or limitations of study protocols. Measurements of energy expenditure require great precision and should be performed in a way that allows analysis during daily activities. As robustness of the generated data depends on equipment quality, respiratory chambers for indirect calorimetry over 24 hours provide more accurate data than commonly used hood systems. Body composition measurements by DEXA are also more accurate than anthropometry or bio-impedance.

Against this background, we hypothesized that gastric bypass in humans results in higher energy expenditure after food intake, *ie* higher postprandial thermogenesis compared to VBG patients. Our secondary outcome was energy expenditure over 24 hours after both surgical techniques. Energy expenditure was measured by indirect calorimetry in a respiratory chamber using weight stable patients that were matched for weight and body composition 9 years after randomisation to laparoscopic Roux-en-Y gastric bypass or laparoscopic vertical-banded gastroplasty [14], [29].

## Methods

### Ethics Statement

The study was approved by the local ethics committee of the University of Gothenburg (Ref 359-09) and was conducted according to the principles of the Helsinki declaration. At inclusion, after having received information both orally and in writing, all patients signed a written consent approved by the local ethics committee.

### Inclusion

Fourteen female patients who previously participated in a randomized clinical trial comparing gastric bypass and VBG (ClinicalTrials.gov ID: NCT01639677, Randomized Clinical Trial Between Laparoscopic Gastric Bypass and Laparoscopic Vertical Banded Gastroplasty for Morbid Obesity) [29] were included 9.4 years (range 8.7–10.3) after surgery to allow us to study patients after long term weight loss maintenance. Many of the patients who had VBG operations were converted to gastric bypass operations and thus there were only seven of the original 34 female VBG patients available to participate in our study. We further included 7 female gastric bypass patients from the same study that primarily matched the VBG group for gender, preoperative body mass index (BMI), preoperative resting energy expenditure and postoperative BMI. All patients were weight stable at least 3 months prior to the study and received similar multivitamin and mineral supplementations for three weeks before the start of investigations. Table 1 summarizes the demographic data for both groups with no significant differences in preoperative BMI, body composition or REE as measured with a canopy indirect calorimeter [14]. Mean age was higher in the gastric bypass group. Postoperatively both the gastric bypass and VBG groups respectively had lowered their BMI from 42.2 (CI 39.2 to 45.2) to 30.8 (CI 27.1 to 34.6) kg/m^2^ (p<0.001) and 43.0 (CI 39.5 to 46.6) to 35.0 (CI 30.7 to 39.3) kg/m^2^ (p<0.001). The pre and postoperative weights did not differ significantly (p = 0.74 and p = 0.29 respectively).

**Table 1:** Demographics and body composition.

	Gastric bypass	VBG	p value
Pre surgery			
Basal metabolic rate [cal/kg/min]	12.1 (11.1–13.1)	11.7 (10.4–13.1)	0.86
BMI [kg/m^2^]	42.2 (39.2–45.2)	43.0 (39.5–46.6)	0.74
Total body mass [kg]	111.2 (101.3–121.0)	113.3 (108–118.6)	0.76
Lean tissue [kg]	49.4 (44.4–54.4)	52.2 (49.7–54.7)	0.43
Lean tissue/total body mass [%]	44.4 (41.8–47.1)	46.2 (44.1–48.2)	0.41
Adipose tissue [kg]	59 (52.7–65.3)	58.1 (53.5–62.7)	0.86
Adipose tissue/total body mass [%]	53 (50.2–55.8)	51.2 (49.1–53.3)	0.41
9.4 y after surgery			
Age on study day [years]	59.7 (54.9–64.4)	50.2 (44.9–55.4)	0.03
BMI [kg/m^2^]	30.8 (27.1–34.6)	35.0 (30.7–39.3)	0.29
Total body mass [kg]	84.2 (72.6–95.9)	92.8 (83.4–102.2)	0.32
Lean tissue [kg]	45.3 (40.9–49.6)	46.4 (44.1–48.8)	0.68
Lean tissue/total body mass [%]	54.1 (51–57.3)	50.6 (46.8–54.5)	0.23
Adipose tissue [kg]	37.2 (29.7–44.6)	43.8 (35.9–51.7)	0.29
Adipose tissue/total body mass [%]	43.4 (40.6–46.2)	46.5 (42.4–50.7)	0.28
Excess BMI loss [%]	67.2 (49.7–84.7)	47.5 (31.2–63.7)	0.16
Total weight loss [kg %]	27.7 (20.1–35.2)	19.8 (13.2–26.4)	0.18
Lean tissue loss [kg]	6.2 (3.4–9)	5.4 (4.1–6.7)	0.69
Adipose tissue loss [kg]	25.5 (16.5–24.5)	16.4 (8–24.7)	0.25

Demographics and body composition in gastric bypass (n = 7) and VBG (n = 7) patients before and 9.4 years after surgery. Values are mean (confidence interval).

### Surgery

Gastric bypass and VBG were performed laparoscopically as previously described [29]. The gastric bypass technique included a small gastric pouch (10–20 mL) connected to the jejunum in an antecolic-antegastric Roux-en-Y construction [30]. The length of the Roux-limb was 75 cm and the entero-entero anastomosis was created 30 cm distal from the ligament of Treitz [30]. The VBG technique included a small gastric pouch (10–20 mL) and a separated vertical staple line [31]. The gastric pouch outlet was reinforced with a 5.0 cm pre-stretched Gore-Tex band [31].

### Preparations

All the patients had a standardized dinner of mashed potatoes and meatballs at 19.00–20.00 the night before the study. The next morning patients arrived at 07.30 after an 11 hour fast. At arrival weight was measured in light underwear. Body mass index (BMI) was calculated and Dual Energy X-Ray Absorptiometry (DEXA) (LUNAR Radiation, Madison, WI, USA) was used to assess total tissue, total adipose tissue, lean body mass, bone mineral density and bone mineral content [32]. Validated food questionnaires were used before surgery and at the study visit to determine the number of calories consumed per day for each patient [33].

### Energy Expenditure

Energy expenditure was measured in an indirect calorimetry chamber for a 24 hour period. The chamber was constructed as a small hotel room measuring 3×3×3 meters. Fresh, dried and filtrated air was circulated, while the temperature and humidity in the chamber was kept constant at 25°C and 40% relative humidity. Oxygen and carbon dioxide contents in the air leaving the chamber were measured constantly enabling an assessment of corresponding energy expenditure of each patient. The chamber was calibrated according to a predefined protocol before each study visit. [34].

**Figure 1:**
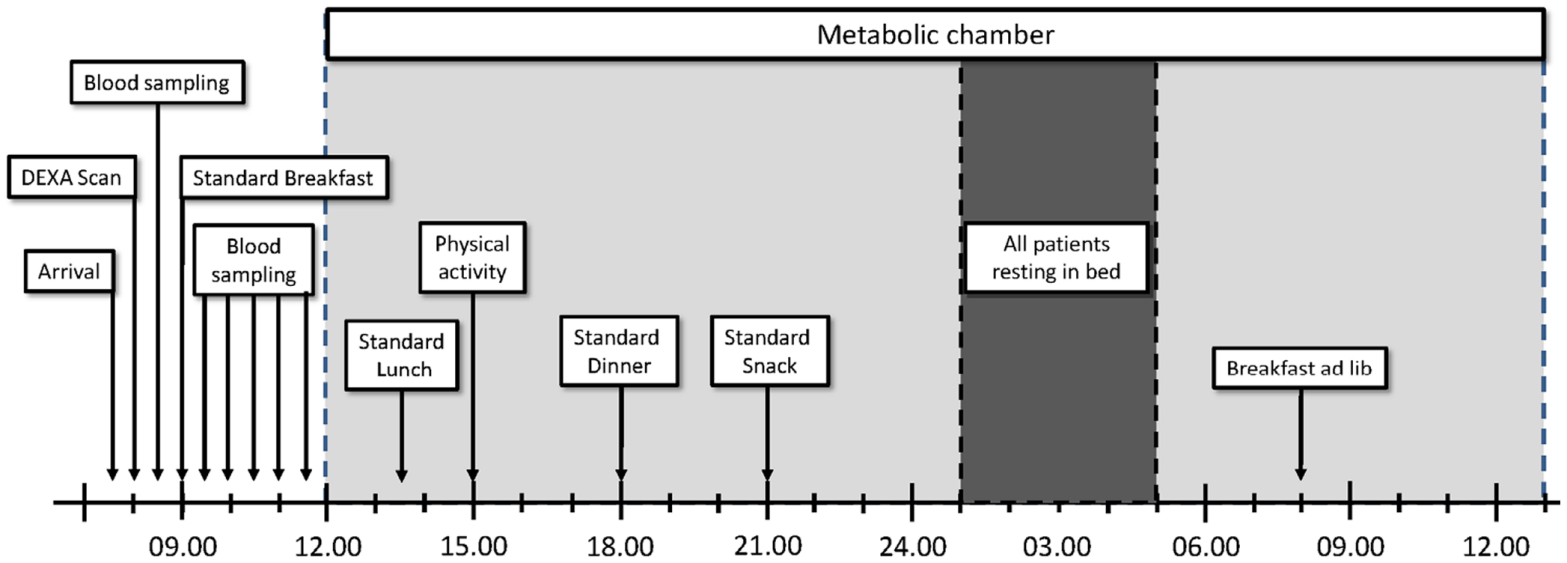
Protocol. Patients arrived at 07.30 after an 11 hour fast. The patients spent 25 hours in total in the chamber for indirect calorimetry.

Figure 1 shows the standardized protocol used for patients when inside the chamber. Patients received four meals consisting of a 400 kcal lunch at 13.30, a 600 kcal dinner at 18.00, one 120 kcal evening snack at 21.00 and an ad libitum breakfast the following morning at 08.00. The first morning (outside of the chamber) and the midday (in the chamber) standard meals consisted of a standard semi liquid meal (400 kcal with 8 E% protein, 36 E% carbohydrate, 56 E% fat) as previously described [9], [12], [35]. Standard dinner consisted of meat balls and mashed potatoes (600 kcal with 13 E% protein, 41 E% carbohydrate, 46 E% fat). The 120 kcal snack consisted of 27 E% protein, 46 E% carbohydrate, 27 E% fat. Patients were asked at 15.00 to cycle for 30 minutes on a stationary ergometry bicycle at 50–55 revolutions per minute. Analyses for energy expenditure (EE) were performed as calories per minutes divided by total tissue in kilogram measured by DEXA (cal_*_min^−1^_*_kg^−1^).

### Energy Expenditure after Food Intake

Energy expenditure measured during the hour before dinner when the patients were awake but in a resting state (17.00–18.00) was used as baseline energy expenditure prior to eating. Energy expenditure after food intake was measured over a two hour period starting when patients initiated dinner (18.00–20.00). The increase in energy expenditure after food intake was calculated as AUC. The delta value was calculated by subtracting resting state energy expenditure (cal_*_min^−1^_*_kg^−1^) from postprandial energy expenditure (cal_*_min^−1^_*_kg^−1^).

### Energy Expenditure during Sleep/Resting Metabolic Rate

Energy expenditure during sleep was measured over 4 hours between 01.00 and 05.00. During this period all patients exhibited a low stable energy expenditure and activity level consistent with sleeping behaviour but not equal to zero. Resting metabolic rate was not assessed in this study.

### 24 Hour Energy Expenditure and Physical Activity

In total, patients spent 25 hours in the chamber. Thirty minutes in the beginning and 30 minutes at the end of the study in the chamber were excluded from analysis to allow for acclimatization, settling after entrance and preparation before exiting. Thus, 24 hours were available for analysis. The predefined exercise requirements were 300 Watt (30 minutes of cycling at 50 to 55 revolutions per minute on the stationary ergometry bicycle). No patient reached the predefined exercise load secondary to joint pains and early exhaustion. A period from when exercise started at 15.00 until all patients returning to pre exercise baseline energy expenditure at 17.45 was therefore excluded from 24 hour energy expenditure analysis.

### Respiratory Quotient (RQ)

Respiratory quotient (RQ) as per definition was calculated from the ratio between CO_2_ eliminated and O_2_ consumed during metabolism. A ratio close to 1 signifies carbohydrate oxidation and a value close to 0.7 is associated with fat oxidation.

### Activity Index

Activity was measured by the number of times patients broke infrared light beams placed strategically in the chamber as described previously [34].

### Blood Samples

At 08.30 fasting blood samples were collected for plasma levels of free fatty acids, high density lipoprotein (HDL), low density lipoprotein (LDL), iron, glycated haemoglobin (HbA1c), free thyroxin (fT4), thyroid stimulating hormone (TSH), follicle stimulating hormone (FSH) and creatinine. While still outside the chamber, the patients were served a 400 kcal standard breakfast at 09.00 (8 E% protein, 36 E% carbohydrate, 56 E% fat) [9], [12], [35] and blood samples were collected prior to and every 30 minutes for 150 minutes postprandially. Plasma glucose, insulin, glucagon-like-peptide 1 (GLP-1) and peptide YY (PYY) were measured [11], [36].

### Statistical Analysis

Data are presented as means (confidence intervals). Differences between groups were evaluated with 2-sample *t* tests where data were normally distributed. The conventional p<0.05 was used as the statistical rejection criterion. IBM SPSS Statistics 20 and Microsoft Excel 2010 were used for statistical analysis.

## Results

### BMI, Body Composition and Food Intake

There were no significant differences comparing the VBG patients included in our study with the complete group of VBG patients in the initial clinical randomized trial between gastric bypass and VBG surgery regarding weight, BMI or age neither preoperatively nor at the time for inclusion in our study (data not shown). Table 1 shows that there were no significant differences in mean BMI, weight loss or excess BMI loss between the gastric bypass and VBG group at the time of energy expenditure measurements. Body composition was also similar between both groups as regards weight, adipose or lean tissue.

**Figure 2:**
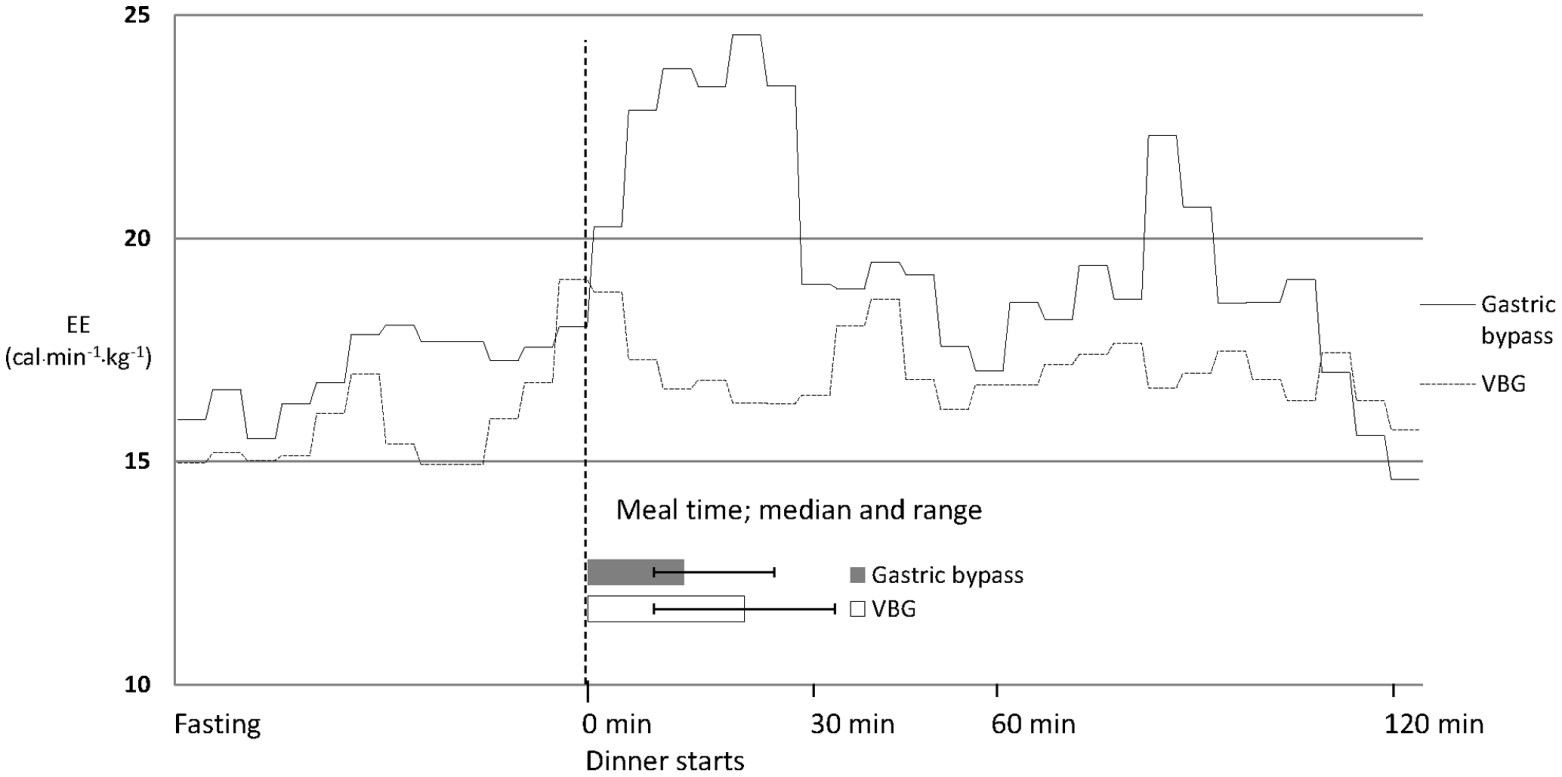
Energy expenditure during dinner intake. Mean energy expenditure (cal⋅min-^1^⋅kg-^1^) one hour before dinner and two hours after dinner started. The duration of the meal is also shown as median and range.

There were no differences regarding total energy intake or intake of macronutrients before or after surgery as estimated by food diaries. Reported mean ad libitum daily energy intake at the time of investigation was 1835 kcal (CI 1614 to 2057) in the gastric bypass group and 1891 kcal (CI 1244 to 2539) in the VBG group (p = 0.89). Figure 2 shows that there was no difference between groups in the time it took to eat dinner, 18.1 (CI 12.8 to 23.5) min for the gastric bypass group and 24.6 (CI 16.7 to 32.5) min for the VBG group (p = 0.21).

### Laboratory Assessments

Table 2 shows that all parameters were within the reference range and there were no significant differences between groups for fasting serum levels of free fatty acids, low density lipoprotein (LDL), iron, HbA1c, free thyroxin (fT4), thyroid stimulating hormone (TSH) or creatinine. Gastric bypass patients had elevated levels of high density lipoprotein (HDL) compared to VBG patients. Plasma samples analysed for GLP-1 and PYY in relation to a standardized meal showed exaggerated responses only in the gastric bypass group.

**Table 2:** Biochemical variables.

	Gastric bypass	VBG	p value
Fasting			
Free fatty acid [mmol/L]	1.02 (0.9–1.14)	1.09 (0.86–1.33)	0.63
HDL [mmol/L]	2.4 (2.1–2.6)	1.5 (1.2–1.7)	0.0007
LDL [mmol/L]	3.5 (2.9–4.2)	3.1 (2.5–3.6)	0.34
Iron [µmol/L]	14.2 (8.1–20.2)	13.3 (6.4–20.2)	0.87
HbA1c [%]	5.2 (4.7–5.6)	4.6 (4.3–4.8)	0.062
Free T4 [pmol/L]	17.7 (14.4–21.0)	15.7 (14.3–17.2)	0.34
TSH [mIU/L]	1.9 (1.3–2.5)	1.7 (1.2–2.2)	0.73
Creatinine [µmol/L]	53.5 (31.1–75.8)	66.8 (60.3–73.3)	0.43
After standard meal; PYY and GLP-1 [pmol/L over 150 minutes]			
PYY AUC	3597.3 (3185.7–4008.4)	1958.4 (1546–2370.8)	0.0002
GLP-1 AUC	6169.1 (5532.2–6805.9)	3394.2 (2696.9–4091.4)	0.0001

Biochemical variables in gastric bypass (n = 7) and VBG (n = 7) patients. Area under the curve (AUC) values of GLP-1 and PYY were calculated using fasting and following a standard 400 kcal meal samples over a 150 minutes period where samples were obtained every 30 minutes. Values are mean (confidence interval).

### Energy Expenditure after Food Intake

Table 3 demonstrates that baseline energy expenditure one hour before dinner intake, when patients were awake but in resting state, was similar in the gastric bypass and VBG patients. However, during two hours after starting to ingest the 600 kcal meal the gastric bypass group had higher energy expenditure in comparison to VBG patients. Moreover, the change from baseline in energy expenditure in the gastric bypass group was greater than that of VBG patients. Energy expenditure was measured after all meals but a significant difference between groups was only seen after dinner which was the meal with the greatest energy content.

**Table 3:** Energy expenditure and respiratory quotient.

	Gastric bypass	VBG	p value
Energy expenditure			
Total 24 hour			
Cal/min/kg total tissue	16.98 (15.54–18.42)	14.92 (14.01–15.83)	0.048
During 1 hour before dinner intake			
Cal/min/kg total tissue	17.04 (15.03–19.06)	15.84 (14.93–16.75)	0.342
During 2 hours after dinner intake			
Cal/min/kg total tissue	19.61 (17.94–21.27)	16.99 (16.1–17.88)	0.019
Delta energy expenditure between fasting and 2 hours after dinner			
Cal/min/kg total tissue	2.56 (1.58–3.55)	1.15 (0.64–1.66)	0.028
Sleep between 01∶00–04∶59			
Cal/min/kg total tissue	12.83 (11.65–14)	11.79 (11.17–12.41)	0.182
Respiratory quotient			
Total 24 hour	0.70 (0.67–0.74)	0.71 (0.67–0.75)	0.931
During 2 hours after dinner	0.77 (0.72–0.81)	0.74 (0.70–0.79)	0.548

Energy expenditure and respiratory quotient for gastric bypass (n = 7) and VBG patients (n = 7) 9.4 years after surgery.

Energy expenditure are analyzed s cal/min/kg total tissue. 24 hour values for GBP and VBG patients were calculated after the 165 minutes post physical activity was excluded. Values are mean (confidence interval).

### Energy Expenditure during Sleep and over 24 Hours

As shown in Table 3 there were no differences in energy expenditure between 01.00 - 04.59 when the patients were sleeping. The mean 24 hours energy expenditure per total tissue mass was however significantly higher for the gastric bypass group compared to the VBG group (p = 0.048). Ad hoc analysis of 24 hours energy expenditure per lean tissue or adipose tissue mass was not significantly different between the groups (p = 0.30 and p = 0.12 respectively).

### Respiratory Quotient (RQ) and Activity Index (AI)

Table 3 demonstrates that over the entire 24 hour period and postprandially there was no difference in respiratory quotient (RQ) in patients after gastric bypass or VBG surgery. The gastric bypass group also had a similar activity index (AI) of 9.3 (CI 6.7 to 12) to the VBG group 7.6 (CI 6.9 to 8.2) (p = 0.24).

## Discussion

Nine years after surgery female patients treated with gastric bypass for morbid obesity had higher energy expenditure immediately (and lasting for at least 2 hours) after intake of a standard meal compared to weight matched patients treated with vertical banded gastroplasty (VBG). Furthermore, higher energy expenditure in the gastric bypass group persisted over 24 hours.

Previous data suggest that gastric bypass as well as other intentional body weight loss methods reduce resting energy expenditure [23], [26], [37]. However, Flancbaum et al’s prospective study with 70 severely obese patients before and 3, 6, 12, 18, and 24 months after surgery demonstrated an increase in REE after gastric bypass in those patients who had a low energy expenditure prior to surgery [28]. These discrepant findings suggest that data must be interpreted with great care especially as studies relied on indirect calorimetry using a canopy system for only 15–20 minutes allowing neither ‘free living’ conditions nor energy expenditure after meal ingestion to be analysed.

Measurement over 24 hours in a respiratory chamber can be regarded as the gold standard for estimating energy expenditure after meals and over time. Based on such measurements in obese women before and after intentional weight loss, reduction in energy expenditure has been estimated to be around 18 to 24 kcal/kg weight loss per day [16], [17]. This effect has been attributed to: reduction in body mass (particularly lean body mass), decrease in postprandial energy expenditure due to less food intake and decreased energy cost of physical activity [16]. Unfortunately, respiratory chamber assessments were not performed prior to surgery in the present study making it impossible to quantify changes in 24 hour energy expenditure over the nine years period. On the other hand, the gastric bypass patients were matched to patients exhibiting similar pre-operative REE as well as similar levels of postoperative weight loss [14].

The main purpose of surgical treatment of obesity is long term weight loss maintenance and amelioration of co-morbidities. We therefore did our study at weight stability after more than 9 years post surgery. The present protocol did not permit detailed analysis of all activities of daily living like walking up stairs etc, but it was evident that pre-prandial as well as sleeping energy expenditure did not differ between the groups. In contrast, early postprandial energy expenditure in the gastric bypass patients was significantly higher compared to the VBG group, which may at least partly explain the observed increase in 24 hour energy expenditure. Our results are consistent with findings obtained previously in gastric bypass operated rats [24], [25]. Furthermore, energy expenditure in rats after other obesity surgery procedures, such as sleeve gastrectomy and gastric banding, revealed no significant effect on energy expenditure [38], [39]. The data indicate that despite similar weight loss to VBG the patients after the gastric bypass had increased energy expenditure in the early postprandial phase, thereby overcoming the general decrease in energy expenditure that usually follows intentional body weight loss [26]–[28].

Until recently, it was thought that body weight loss after gastric bypass was mainly caused by the combination of mechanical restriction and malabsorption [40]. However, there is a growing body of evidence supporting that other mechanisms such as reduced hunger [10], [12], [41], increased satiety [10], [12], altered taste [42], [43], as well as reduced preference for high caloric dense foods [14], [44]–[46] are responsible for weight loss induction and maintenance after gastric bypass. However, the mechanisms mediating higher postprandial energy expenditure after gastric bypass have not yet been determined. Exclusion of the proximal small bowel from nutrients, and/or the reduction in common channel length may play a role. If so, a similar effect can be expected after biliopancreatic diversion/duodenal switch. The altered anatomy after gastric bypass surgery may change gastrointestinal and central neuroendocrine signalling after food ingestion, which in turn may induce the increase in energy expenditure. Postprandial release of bile acids and gut hormones such as PYY, oxyntomodulin and GLP-1 are markedly enhanced after gastric bypass and may influence total energy expenditure [26], [29], [42]. Our findings that postprandial PYY and GLP-1 responses are still increased nine years following gastric bypass surgery are consistent with the before mentioned hypothesis.

The present study has several limitations: Energy expenditure prior to surgery was not measured in a 24 hour chamber, but with an indirect calorimeter using a canopy. Although the study only tested patients on one occasion, 9.4 years after surgery the patients were weight stable, healthy and were asked to continue their physical activity, work, sleep and eating habits the week prior to the study. Even though patients were recruited from a randomized controlled trial, the small sample size and the fact that we only studied female patients may limit generalizability. The small sample size may also explain why we did not see a difference in 24 hour energy expenditure when adjusted for lean body mass. We were not able to measure post-prandial thermogenesis for more than 2 hours even though it has been shown to remain elevated up to 5 hours after a meal [16] in resting subjects. Our patients consumed 120 kcal after 2 hours, because the meal frequency of patients after RYGB increases and patients usually eat smaller but more frequent meals [13]. The protocol used for this study therefore tried to reflect the usual eating pattern of patients after surgery with a small snack 2 hours after dinner which unfortunately prohibited the measurement of postprandial energy expenditure over 5 hours after the evening meal. All patients were asked to exercise during their stay in the chamber, but none of them was able to achieve the predefined physical activity which necessitated exclusion of this period from analysis. Finally, the gastric bypass group exhibited a (non-significant) larger body weight loss and higher age than the VBG group. However, both these results would, if anything, mediate a greater reduction in energy expenditure as oppose to an enhanced energy expenditure as we observed in the gastric bypass group [26], [29], [30].

In summary, the present study indicates that patients treated with gastric bypass had higher early postprandial energy expenditure compared to patients having similar weight loss following a vertical banded gastroplasty. The postprandial effect partly explains why 24 hour energy expenditure is higher in gastric bypass patients and why this operation is superior to vertical banded gastroplasty regarding long-term weight loss maintenance. The yet to be determined gastrointestinal mechanism(s) activating energy expenditure in association with gastric bypass may offer novel therapeutic targets for obesity treatments or allow for better and safer surgical operations to be designed.
